# Prognostic Significance of PD-L1 Expression In Patients With Primary Oropharyngeal Squamous Cell Carcinoma: A Meta-Analysis

**DOI:** 10.3389/fonc.2021.787864

**Published:** 2021-11-25

**Authors:** Jerry Polesel, Anna Menegaldo, Giancarlo Tirelli, Vittorio Giacomarra, Roberto Guerrieri, Lorena Baboci, Mariateresa Casarotto, Valentina Lupato, Giuseppe Fanetti, Paolo Boscolo-Rizzo, Elisabetta Fratta

**Affiliations:** ^1^ Unit of Cancer Epidemiology, Centro di Riferimento Oncologico di Aviano (CRO), IRCCS, Aviano, Italy; ^2^ Unit of Otolaryngology, AULSS 2 - Marca Trevigiana, Treviso, Italy; ^3^ Department of Medical, Surgical and Health Sciences, Section of Otolaryngology, University of Trieste, Trieste, Italy; ^4^ Division of Otolaryngology, General Hospital “S. Maria degli Angeli”, Pordenone, Italy; ^5^ Division of Immunopathology and Cancer Biomarkers, Centro di Riferimento Oncologico di Aviano (CRO), IRCCS, Aviano, Italy; ^6^ Division of Radiotherapy, Centro di Riferimento Oncologico di Aviano (CRO), IRCCS, Aviano, Italy

**Keywords:** oropharyngeal squamous cell carcinoma, head and neck squamous cell carcinoma, PD-L1, HPV, prognostic biomarkers

## Abstract

**Background:**

At present, the prognostic significance of programmed cell death receptor ligand 1 (PD-L1) expression in oropharyngeal squamous cell carcinoma (OPSCC) patients is still controversial. In this study, we aim to synthesize relevant studies that have assessed the prognostic value of PD-L1 in patients with primary OPSCC treated according to the current standard-of-care.

**Methods:**

A systematic search of Medline/PubMed, Cochrane, Embase, Web of Science, and Scopus was conducted to define the prognostic role of PD-L1 expression in OPSCC. All studies published before July 31, 2021 were screened. Summary hazard ratios (sHR) with 95% confidence intervals (CIs) were calculated using a random-effects model.

**Results:**

A total of 1522 OPSCC patients from 12 studies were included. PD-L1 expression in OPSCC tumor cells (TCs) was significantly associated with longer overall survival (sHR=0.63, 95% CI 0.50-0.79), and progression-free survival (sHR=0.62, 95% CI 0.49-0.79). A benefit in survival was also observed in PD-L1-positive OPSCC patients who underwent surgery (sHR=0.34, 95% CI 0.18-0.65). Finally, although PD-L1-positive expression was related to better outcomes both in HPV-negative and HPV-positive OPSCC, the difference reached the statistical significance only in the HPV-positive subgroup (sHR=0.37, 95% CI 0.19-0.73). No heterogeneity emerged between studies for all considered outcomes, with *I* ^2^ ranging from 0% for progression-free survival to 11% for overall survival.

**Conclusions:**

PD-L1 expression on TCs associated with improved survival in OPSCC. In particular, HPV-positive OPSCC most benefited from PD-L1 expression when compared to the PD-L1 negative counterpart. Thus, PD-L1 might represent a useful biomarker to stratify prognosis in OPSCC in addition to HPV status.

## Introduction

Despite its high immunogenicity, tumor microenvironment (TME) of both primary and recurrent head and neck squamous cell carcinoma (HNSCC) is associated with pronounced immunosuppressive activity (1). Immunosuppression creates an advantageous environment for HNSCC cells, that can evade tumor immune surveillance through various strategies, including the recruitment of suppressive cell populations, release of tumor-derived soluble immunosuppressive factors, up-regulation of immune checkpoint inhibitors (ICI), and impaired co-stimulatory signaling (2).

At present, the up-regulation of the transmembrane glycoprotein programmed death ligand 1 (PD-L1) (3) is among the most potent and thoroughly investigated strategy implemented by tumor cells (TCs) and TME to suppress cellular immune responses. Once expressed, PD-L1 binds to the programmed death 1 (PD-1), a negative co-stimulatory receptor that inhibits the activation of a wide range of immune cells, including peripherally activated T cells, B cells, monocytes, natural killer cells, and certain dendritic cells, thus playing a crucial role in the maintenance of immune tolerance of self-antigens (4, 5). Blocking these ICI has become an important direction of immunotherapy with antibodies targeting PD-1 or PD-L1 being currently approved for the treatment of multiple cancers including HNSCC (6). In 2016, the PD-1 inhibitors pembrolizumab and nivolumab have been approved by the Food and Drug Administration (FDA) for use in patients with recurrent or metastatic (R/M) HNSCC who progressed on standard platinum-based therapy (7). More recently, FDA extended the indications for pembrolizumab monotherapy in the first-line treatment of R/M HNSCC patients whose tumor and/or immune cells expressed PD-L1 with a Combined Positive Score (CPS) ≥1 (8). Therefore, the ICI-based therapy blockade has been rapidly progressing in HNSCC, with a survival benefit in approximately 20-30% of patients (9–11).

Among HNSCC, the TME of the subset of oropharyngeal SCC (OPSCC) caused by human papilloma virus (HPV) is more enriched in immune cells than the HPV-negative counterpart, harbors higher levels of immune activation, and is characterized by the presence of cytotoxic T lymphocytes (CTL) specifically directed against HPV16 E6 and E7 oncoproteins (12, 13). Furthermore, a more pronounced expression of the immunoinhibitory receptors including CTLA-4 and PD-1 has been observed in HPV-positive OPSCC (14, 15). Thus, a highly immunogenic and more strongly immune infiltrated TME seems to be countered by a greater involvement of immunosuppressive strategies. These observations suggest that HPV-positive patients are more likely to benefit from anti-PD-1/PD-L1 therapy respect to HPV-negative ones. However, the current standard-of-care treatment for patients with primary OPSCC is still surgery +/- adjuvant (chemo)radiotherapy or up-front (chemo)radiation with the last being a preferred option in several cases (16).

Recent studies have showed that radiotherapy can activate tumor-specific immune responses and these responses may be modulated by immune landscape of the TME (17). Moreover, PD-1 and PD-L1 expression was observed to predict radiosensitivity in HNSCC (18). Therefore, in addition to evaluating PD-1/PD-L1 expression as predictor of response to immunotherapy, there has been equally interest in evaluating the prognostic role of PD-1/PD-L1 in patients with primary OPSCC treated according to the current standard-of-care. A recent meta-analysis reported that HNSCC patients expressing PD-L1 may have a better tumor response and overall survival (OS) irrespective of their HPV status (19). However, data regarding the prognostic role of PD-L1 in HPV-positive OPSCC are still limited and controversial. In fact, although many studies have found that PD-L1 expression on TCs was associated with favorable prognosis of HPV-positive OPSCC patients (20–27), others found no association with prognosis or even a reverse relationship (28–35). Notably, the diverging results found in these studies may possibly depend on the use of different anti PD-L1 antibodies and cut-off values for determining PD-L1 positivity.

Thus, given the inconsistency and inconclusive findings of the published data, we carried out an up-to-date systematic review and meta-analysis to determine whether PD-L1 expression affects OS, progression-free survival (PFS) and/or loco-regional control (LRC) of patients with primary HPV-positive and HPV-negative OPSCC treated according to the current standard-of-care.

## Materials and Methods

### Ethics Statement

Data from this meta-analysis were based on previous published papers; therefore, this study did not require ethical approval or patient consent.

### Outcome Measures

The primary outcome of this meta-analysis was to investigate the prognostic value of PD-L1 for OS (defined as the time from diagnosis or initiation of treatment to patient death, irrespective of cause), PFS (defined as the time from diagnosis or initiation of treatment until tumor recurrence/progression or any-cause death), and LRC (defined as the time from diagnosis or initiation of treatment to the first loco-regional event) in patients treated by upfront surgery or (chemo)radiotherapy for OPSCC.

The secondary endpoint was the prognostic significance of PD-L1 on the above clinical outcome variables stratifying patients by type of treatment, upfront surgery or upfront (chemo) radiotherapy, and HPV status, as detected by p16 immunohistochemistry (IHC), HPV-DNA *in situ* hybridization or PCR.

### Search Strategy

**Figure 1:**
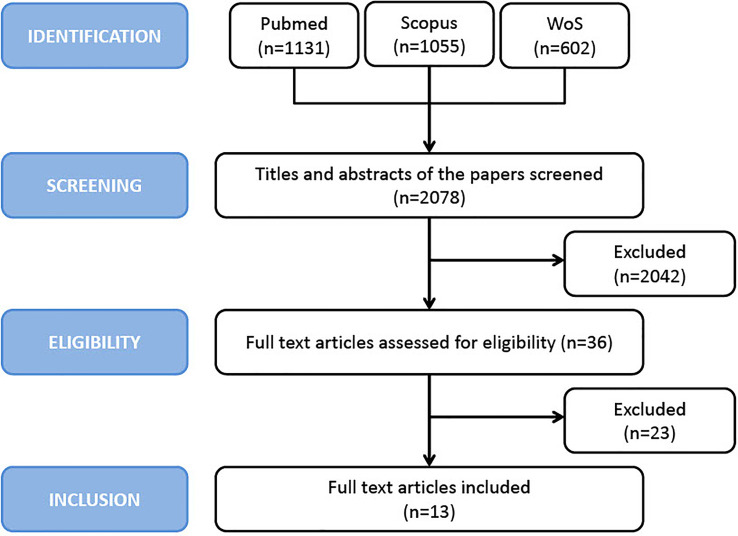
PRISMA flow chart of study inclusion process.

This systematic review and meta-analysis were conducted following the preferred reporting items for systematic reviews and meta-analyses (PRISMA) checklist. Medline/PubMed (*via* Ovid), Cochrane, Embase (*via* Ovid), Web of Science (Core Collection) and Scopus were searched from inception through the end of July 2021. The research was conducted according to PRISMA criteria (36). The following keyword search was conducted: “head and neck” OR “facial” OR “mouth” OR “oral cavity” OR “oropharyngeal” OR “pharyngeal” OR “oropharynx” OR “pharynx” AND “squamous cell carcinoma*” OR “carcinoma*” OR “tumor*” OR “cancer*” OR “neoplasm*” AND “PD-L1” OR “B7-H1” OR “CD274” AND “prognosis” OR “risk” OR “recurrence*” OR “mortality” OR “survival” OR “outcome*”. The reference lists of articles included in this review as well as narrative reviews published in the last 10 years were also manually searched to minimize the risk of missing data. Two authors (PBR and EF) independently screened all titles and abstracts generated by the search and then evaluated the full texts of all the relevant articles identified against the inclusion criteria (**Figure 1**); a third author (JP) settled discordances when present. Any disagreement between the assessors on the suitability of articles for inclusion were tackled thorough discussion between assessors, or failing this, by referral to the other authors.

### Selection Criteria

Studies were included in the analysis if they met the following criteria: 1) the study reported the prognostic role of PD-L1 in primary non-metastatic OPSCC; 2) the patients were treated according to the current standard of care; 3) studies in which PD-L1 expression was detected by IHC; 4) the study reported the association of PD-L1 and patient outcome with sufficient survival data to extract hazard ratios (HRs) and 95% confidence intervals (CI). Non-English studies were excluded. Studies containing aggregated data or duplicated data from previously published work were excluded, as were review articles, case reports, editorials, and letters. Two authors (JP, PBR) independently assessed the quality of the included studies with the Newcastle-Ottawa Scale (37). Low-quality articles (Newcastle–Ottawa Scale (NOS) score <4) were also excluded.

### Data Extraction and Statistical Analysis

The standard error of the log hazard ratio was derived from the log CIs. The summary HR (sHR) and corresponding 95% CI were calculated according to random-effects models of DerSimonian and Laird (38), which incorporates both within-and between-study variability, as a weighted average of the estimated HRs, by giving each study a weight proportional to its precision. Statistical heterogeneity among studies was evaluated using the *I*² and τ² statistics (38). Influence analysis was performed when sHRs were estimated from five or more studies: sHR was calculated by omitting one study at a time. Publication bias was assessed through a funnel plot (39). The results of the meta-analysis were presented graphically using forest plots, plotting the individual paper and sHR with corresponding 95% CI. Statistical significance was defined as p <0.05 (two sided).

## Results

### Search Results and Study Characteristics

A total of 2078 potentially relevant articles were identified with our initial search strategy. After screening the titles and abstracts of these articles, 2042 studies were excluded because they were deemed repetitive or unqualified. After reading 36 potentially eligible articles in detail, 13 studies met our inclusion criteria. However, since the study by Salomon et al. (27) excluded patients with HPV-negative OPSCC, thus introducing a potential selection bias, it was excluded from the analysis, thus leaving 12 articles in the final analysis (17, 20–25, 28–30, 32, 34). No additional studies were obtained by checking the reference lists of these articles. **Figure 1** presents a detailed diagram of the above screening process.

The characteristics of the included studies are reported in **Table 1**. The sample sizes of these studies ranged from 65 to 303 patients for a total of 1522 patients. Different cut-off values for the percentage of PD-L1-positive TCs were utilized in 12 studies (17, 20–25, 27–30, 34), ranging from 1% to 50%, while one study used the Combined Positive Score (CPS) ≥1 to define a PD-L1 positive OPSCC (32). The rates of PD-L1 positive OPSCC in the above studies ranged from 7.7% to 88.7% with overall 910 patients (53.4%) being PD-L1 positive. With the exception of one study that only selected HPV positive OPSCC (27), all other studies included consecutive patient series selected regardless of HPV status.

**Table 1:** Description of included studies.

Study	Country	Enrolment	n	HPV-status	PD-L1 antibody	Cut-off for PD-L1 positivity
Gurin (29)	Czech Republic	–	65	Pos/Neg	Clone 28-8 (Abcam)	TC ≥ 5%
Jeong (30)	Korea	2006-2013	106	Pos/Neg	Clone SP263 (Ventana)	TC ≥ 50%
Lilja-Fisher (32)	Denmark	2000-2012	303	Pos/Neg	Clone 22C3 (PharmDx)	CPS ≥ 1
Hong (22)	Australia	–	214	Pos/Neg	Clone E1L3N (CST)	TC ≥ 1%
Sato (26)	Japan	2000-2016	137	Pos/Neg	Clone E1L3N (CST)	TC ≥ 5%
Fukushima (21)	Japan	2005-2016	92	Pos/Neg	Clone SP142 (Ventana)	TC ≥ 1%
Kwon (25)	South Korea	1997-2010	79	Pos/Neg	Clone SP142 (Ventana)	TC ≥ 5%
Steuer (24)	U.S.A.	1994-2008	97	Pos/Neg	(CST)	TC ≥ 1+
Balermpas (28)	Germany	2004-2012	98	Pos/Neg	Clone E1L3N (CST)	TC ≥ 5%
De Meulenaere (20)	Belgium	2004-2013	99	Pos/Neg	Clone SP142 (Roche)	TC ≥ 5%
Hong (23)	Australia	–	99	Pos/Neg	Clone E1L3N (CST)	–
Kim (24)	South Korea	2002-2013	133	Pos/Neg	B7H1 Clone 5H1	TC ≥ 20%

NOS, Newcastle-Ottawa Scale.

### Quality Assessment

All included studies reported a satisfactory quality (Newcastle­Ottawa Scale score ≥6), with a median of 8 (**Table 2**). The most frequent sources of potential bias were specific selection criteria that could have impaired external validity (e.g., restriction to tonsillar cancer or to patients undergoing radiation therapy) and the lack of reporting information of the completeness of follow-up. Only two studies did not report multivariable estimates.

**Table 2:** Quality assessment of included studies according to the Newcastle-Ottawa Scale (http://www.ohri.ca/programs/clinical_epidemiology/oxford.asp).

Study	Selection				Comparability^a^	Outcome			Total Score (0–9)
	Representativeness of exposed cohort	Selection of non-exposed cohort	Exposure ascertainment	Outcome not present prior to exposure		Independent assessment	Adequacy of follow-up (median ≥24 months)	Completeness of follow-up ascertainment	
Gurin (29)	○	●	●	●	○●	●	●	○	6
Jeong (30)	●	●	●	●	●●	●	●	○	8
Lilja-Fisher (32)	○	●	●	●	●●	●	●	○	7
Hong (22)	●	●	●	●	●●	●	●	○	8
Sato (26)	●	●	●	●	●●	●	●	○	8
Fukushima (21)	○	●	●	●	●●	●	●	●	7
Kwon (25)	○	●	●	●	●●	●	●	●	8
Steuer (24)	●	●	●	●	●●	●	●	○	8
Balermpas (28)	○	●	●	●	●●	●	●	○	7
De Meulenaere (20)	●	●	●	●	○○	●	●	●	7
Hong (23)	○	●	●	●	●●	●	●	●	8
Kim (24)	○	●	●	●	●●	●	●	●	7

a 1 point for multivariable analyses; 1 point for stratification by HPV.

### PD-L1 Expression as a Biomarker of Outcomes

**Figure 2:**
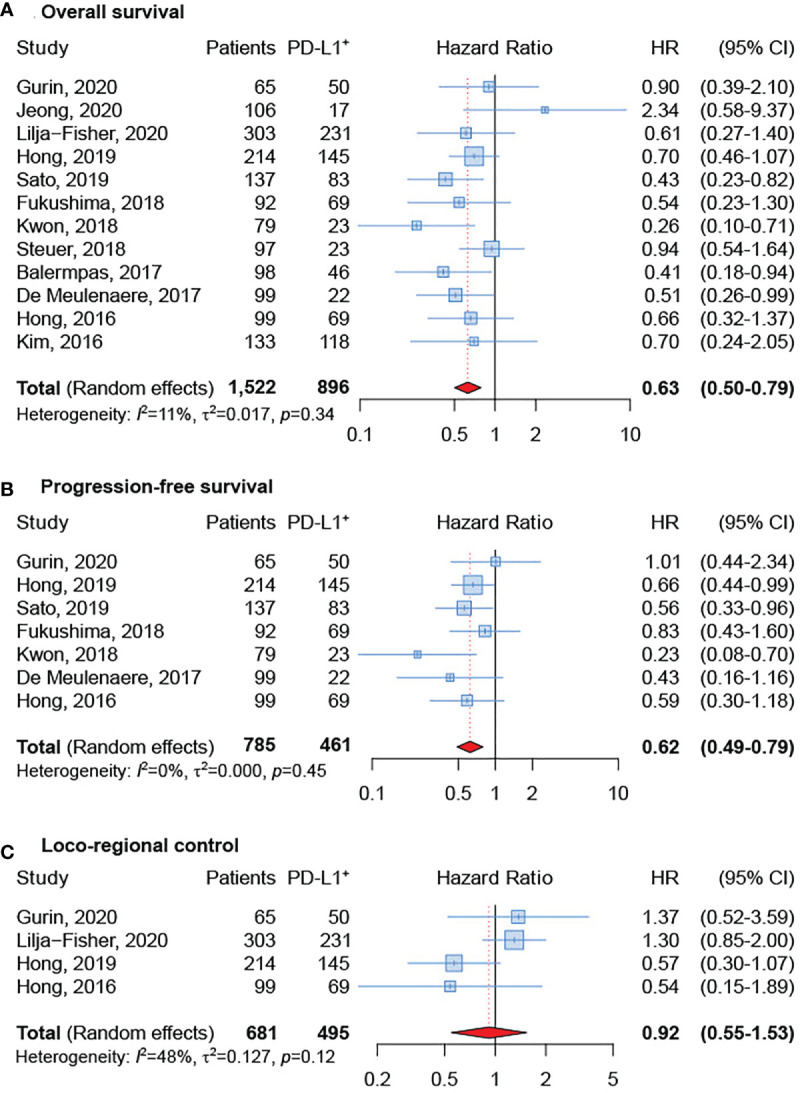
Forest plot for the association between PD-L1 expression and overall survival **(A)**, progression-free survival **(B)**, and loco-regional control **(C)**.

All articles provided HRs and 95% CIs of OS in OPSCC. As shown in **Figure 2A**, no evident heterogeneity was observed in these studies (*I* ^2 =^ 11%; p = 0.34). The results revealed that positive PD-L1 expression was related to a significantly improved OS in patients with OPSCC (sHR = 0.63, 95% CI: 0.50-0.79). Differently, only seven articles provided HRs and 95% CIs for PFS in OPSCC (17, 20–23, 25, 29). As depicted in **Figure 2B**, there was no significant heterogeneity among these 7 articles (*I* ^2 =^ 0%; p = 0.45). The meta-analysis indicated that positive PD-L1 expression was related to greater PFS in patients with OPSCC (sHR = 0.62, 95% CI: 0.49–0.79). Finally, the association between PD-L1 expression and LRC was investigated in four studies (22, 23, 29, 32) and a significant heterogeneity was not detected among them (*I* ^2 =^ 48%; p = 0.12) (**Figure 2C**). Although a significant relationship did not emerged, results showed that LRC was improved in OPSCC patients that were PD-L1 positive (sHR = 0.92, 95% CI: 0.55–1.53).

### Impact of HPV Status

**Figure 3:**
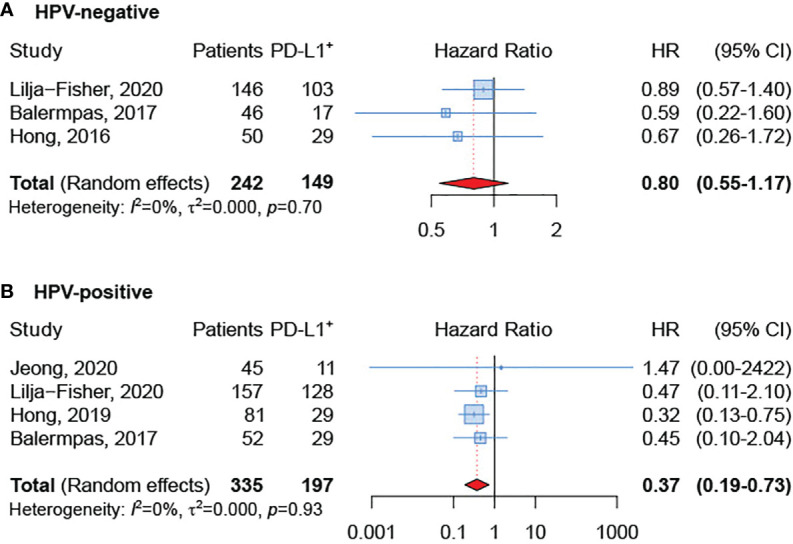
Forest plot for the association between PD-L1 expression and overall survival in HPV-negative **(A)** and HPV-positive **(B)** tumors.

The subgroup analysis based on HPV status showed that PD-L1-positive expression was related to better OS both in HPV-negative and HPV-positive OPSCC. However, few studies (22, 23, 28, 30, 32) reported HRs and 95% CI for OS according to HPV-status and the difference reached the statistical significance only in the subgroup of HPV-positive OPSCC (sHR=0.37, 95% CI 0.19-0.73) (**Figures 3A, B**).

### Impact of Treatment

**Figure 4:**
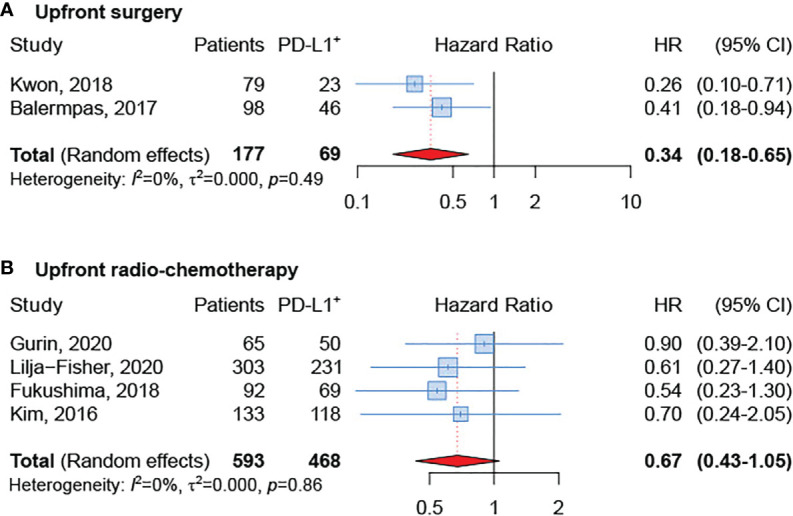
Forest plot for the association between PD-L1 expression and overall survival in patients undergoing upfront surgery **(A)** or upfront radio-chemotherapy **(B)**.

The subgroup analysis based on treatment modality [upfront surgery or upfront (chemo)radiotherapy] revealed a better OS in PD-L1 positive tumors, regardless of treatment type (**Figures 4A, B**). Nevertheless, the effect was greater and significant only in the group treated with up-front surgery (sHR = 0.34, 95% CI: 0.18–0.65). Unfortunately, data available for the present analysis was limited since only six studies were included (21, 24, 25, 28, 29, 32).

### Publication Bias and Sensitivity Analysis

**Figure 5:**
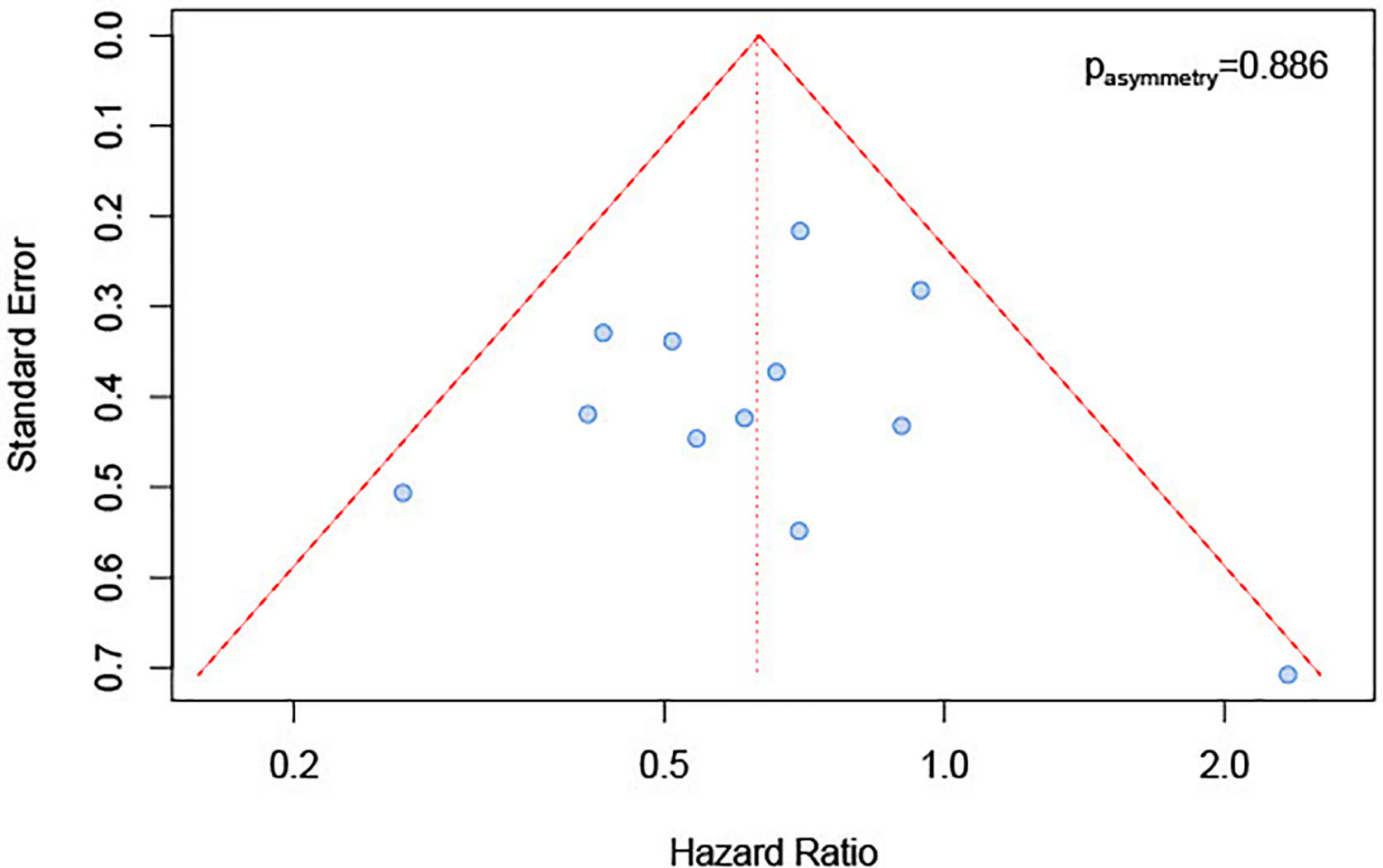
Funnel plot for publication bias.

**Figure 6:**
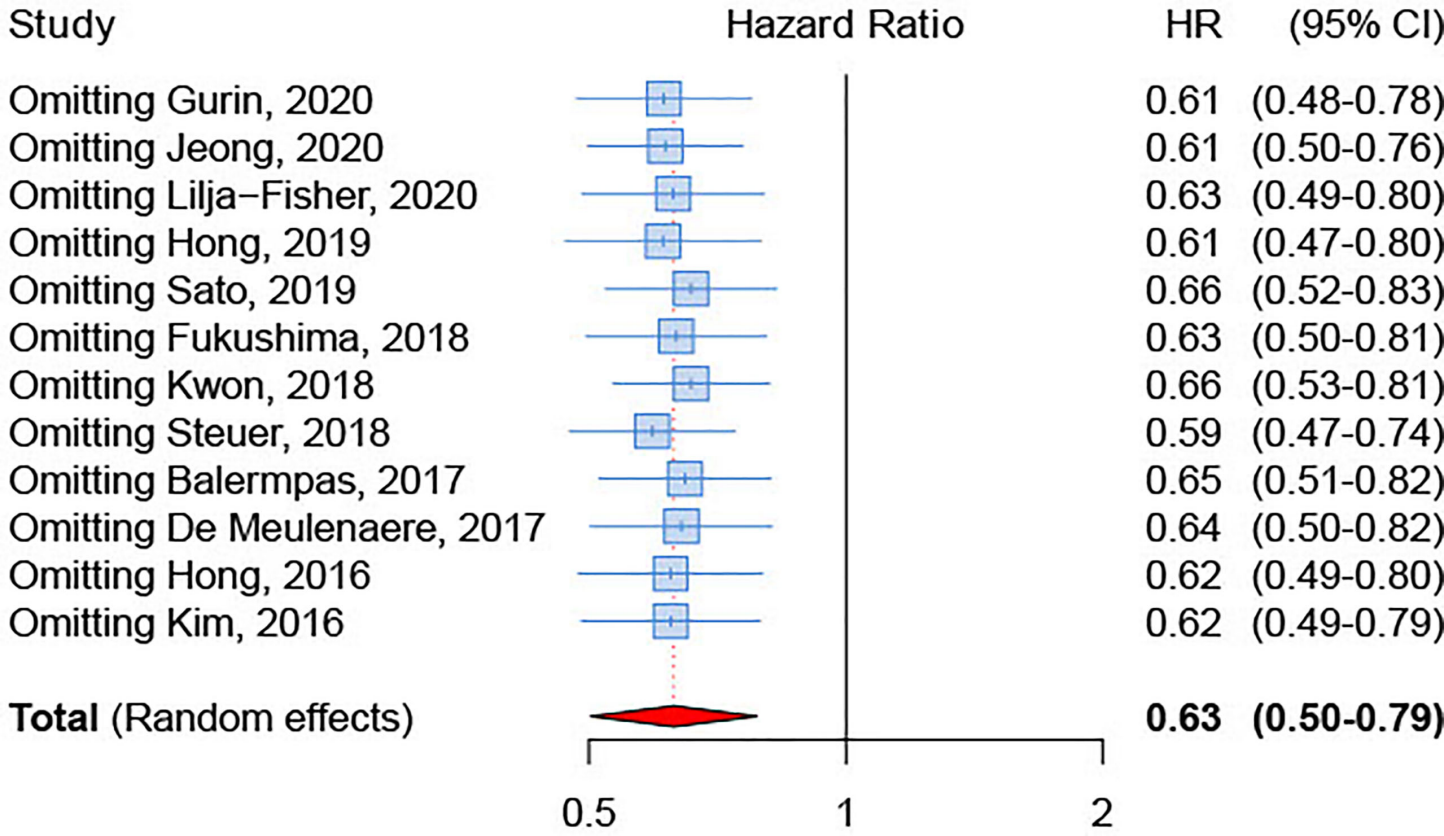
Influence analysis on the association between PD-L1 expression and overall survival.

We inspected publication bias through a funnel plot. The result (**Figure 5**) indicated a lack of publication bias (test for asymmetry in funnel plot: p=0.89). Furthermore, influence analysis was implemented by eliminating one by one studies to determine whether a single study could significantly influence the summary result. The results showed no relevant impact on the overall result by any study (**Figure 6**), with sHRs ranging from 0.59 by excluding Steuer et al. to 0.66 by excluding either Sato et al. (26) or Kwon et al. (25).

## Discussion

In the last years, several studies evaluated PD-L1 expression in HNSCC tissues (40, 41). Nevertheless, the relationship between PD-L1 expression and the prognosis of OPSCC patients was still controversial. Two meta-analyses conducted in 2017 and 2018 have not observed a prognostic effect of tumor PD-L1 positivity in patients diagnosed with OPSCC (42, 43). However, although seventeen studies were considered eligible by Li et al. in 2017, only two of them focused on OPSCC (44). In 2018, Yang et al. 2018 included three additional studies, which mainly comprised OPSCC, but a statistically significant benefit in OS was still not observed (43), thus suggesting these results might depend on the limited number of survival data. The results of this meta-analysis confirmed that, in patients diagnosed with OPSCC, PD-L1 expression was associated with a better OS and PSF, but not with LRC. Notably, higher PD-L1 expression was correlated with improved OS rates in other tumor types, including melanoma (45), colorectal cancer (46), Merkel-cell carcinoma (47), and endometrial cancer (48, 49).

Of interest, in our study, the favorable effect of PD-L1 was stronger for HPV-positive patients. In fact, subgroup analysis by HPV status showed a 60% reduction in mortality in patients who expressed PD-L1 compared to those who did not. Consistent with these data, PD-L1 levels were significantly higher in HPV-positive OPSCC, suggesting a relationship between HPV status and PD-L1 expression. Although HPV oncoproteins E5 and E6/E7 were supposed to activate the PD-1/PD-L1 axis (50), further research is required to elucidate the association between HPV oncoproteins and PD-L1. In addition, since PD-L1 gets up-regulated in response to tumor-infiltrating lymphocytes (TIL)-derived cytokines, the increase in PD-L1 expression in HPV-positive OPSCC might be related to a more inflamed tumor microenvironment with recruitment of TILs, rather than to a direct causative effect of HPV on PD-L1 expression (51). This theory is in line with the evidence that abundant TILs and high PD-L1 expression in intratumoral immune cells identified subgroups of HPV-positive OPSCC patients with excellent outcomes (27). These observations might seem a paradox since TILs are supposed to mount anti-tumor immune responses whereas PD-L1 is assumed to impair their activity. Thus, the impression is that the expression of PD-1/PD-L1 axis may be the reflection of the antitumor reactivity rather than a sign of immune exhaustion. Recent studies have indicated that evaluation of PD-L1 protein status could assist in the selection of patients with OPSCC who are candidates for ICI immunotherapy (52). However, the role of ICI in platinum-refractory recurrent/metastatic (R/M) HNSCC is still controversial with only a small fraction of patients experiencing clinical benefit from anti-PD-1 monotherapy or in combination with chemotherapy (53). On this ground, our findings would also support the use of antibodies targeting the PD-1/PD-L1 axis for these patients. Particularly, the tumor microenvironment of HPV-positive OPSCC, highly infiltrated by immune cells whose activity is blocked by the expression of ICI, makes these tumors ideal candidates for trials testing first-line ICI immunotherapy.

HPV-driven OPSCC have significantly better survival rates than tobacco and alcohol induced HNSCC (54). Since patients with HPV-driven cancer are younger, healthier and far more likely to survive their disease, long-term treatment-related toxicities are major issue in this population. With the aim to reduce toxicity while maintaining efficacy, treatment de-escalation strategies are currently investigated in several clinical trials. However, the only two phase III de-intensification trials failed to show the equivalence of the de-escalated treatment arm (55, 56). One possible explanation may lie in the fact that HPV-positive OPSCC is actually a heterogeneous disease. Interestingly, a subgroup of patients with confirmed HPV-driven OPSCC were shown to have an immunologically “colder” microenvironment and markedly poorer clinical outcome (57). Given that PD-L1 is able to stratify the prognosis in the group of HPV-positive tumors, it could be interesting to explore the opportunity of using PD-L1, possibly together with other clinical and molecular parameters, as a tool to improve the selection of patients for treatment de-intensification trials.

### Strengths and Limitations

The strength of our study relies on the inclusion of very recent data, adding further precision to the evidence that PD-L1 expression in TCs might be a favorable prognostic marker in OPSCC. However, several limitations exist in our meta-analysis. First, since the number of included studies was small for some outcomes and for sub-group analyses, bias may have been introduced. This occurred especially when the studies were greatly unbalanced in sample size, as summary estimates were more sensitive to single studies. Further, heterogeneity estimation was challenging in this context; in addition to imprecision, the *I* ^2^ statistic may also suffer of estimation bias (58), claiming for caution in its interpretation. Second, some information was not available in all included studies; for instance, data collected from published papers often lacked individual results for HPV status and/or treatment modality; hence, we failed to conduct a stratified analysis of the influence of PD-L1 expression on the response to surgery and/or radio-therapy in subgroups of HPV-positive and -negative OPSCC patients. In addition, since it has recently been shown that the level of PD-L1 expression was discrepant between primary HNSCC and corresponding distant metastases (59), only primary OPSCC have been considered. Third, this meta-analysis might not include studies that were not published due to negative results, which are often rejected or not even submit. Nonetheless, no publication bias emerged according to funnel plot inspection. Fourth, to compare OPSCC patient survival based on PD-L1 expression status might depend on the variability of IHC assay. In fact, accurate measurement of PD-L1 protein levels in FFPE tumor samples could be problematic due to antibodies discrepancy, differences in types of platforms used for the IHC staining and scoring criteria. A significant degree of intratumoral heterogeneity of PD-L1 expression might also represent an important limitation (60). Finally, the studies included in this meta-analysis have utilized a variety of techniques for determining the HPV status, including p16 IHC, HPV-DNA *in situ* hybridization or PCR. Hence, a standardized method for quantifying PD-L1 expression and determining HPV status is urgently needed.

### Conclusions

The present meta-analysis showed that PD-L1 expression, as measured by IHC, was significantly associated with a better outcome in primary OPSCC treated according to the current standard of care. Particularly, HPV-positive OPSCC most benefited from PD-L1 expression with those expressing PD-L1 showing a reduction of 63% in the risk of death compared to the PD-L1 negative counterpart. Thus, PD-L1 might represent a useful biomarker, outside the context of ICI immunotherapy, to stratify prognosis in OPSCC in addition to HPV status.

## Data Availability Statement

Publicly available datasets were analyzed in this study. This data can be found here: Data are available in the originally published articles.

## Author Contributions

JP, PB-R and EF designed the study and wrote the manuscript. AM, GT, VG, RG, LB, MC, VL and GF critically revised the manuscript. All authors contributed to the article and approved the submitted version.

## Funding

This work was supported by Ministero della Salute Ricerca Corrente and 5x1000 Intramural Grant from Centro di Riferimento Oncologico Aviano (CRO) IRCCS.

## Conflict of Interest

The authors declare that the research was conducted in the absence of any commercial or financial relationships that could be construed as a potential conflict of interest.

## Publisher’s Note

All claims expressed in this article are solely those of the authors and do not necessarily represent those of their affiliated organizations, or those of the publisher, the editors and the reviewers. Any product that may be evaluated in this article, or claim that may be made by its manufacturer, is not guaranteed or endorsed by the publisher.
